# Supplementary material for: Critical roles of metal–ligand complexes in the controlled synthesis of various metal nanoclusters
Source: Nat Commun. 2023 Jun 2;14:3201. doi: 10.1038/s41467-023-38955-y (PMC10238426; doi:10.1038/s41467-023-38955-y)
Supplement: Supplementary file 1 — Supplementary Information [file 41467_2023_38955_MOESM1_ESM.pdf]

Supplementary Information

Critical roles of metal-ligand complexes in the controlled synthesis of various metal nanoclusters

Ji Soo Kim[1,2], Hogeun Chang[1,2,5], Sungsu Kang[1,2], Seungwoo Cha[2,6], Hanguk Cho[1,2], Seung Jae Kwak[2], Namjun Park[2], Younhwa Kim[1,2], Dohun Kang[1,2,7], Chyan Kyung Song[1,2], Jimin Kwag[1,2], Ji-Sook Hahn[2], Won Bo Lee[2], Taeghwan Hyeon[1,2*] and Jungwon Park[1,2,3,4*]

[1] Center for Nanoparticle Research, Institute for Basic Science (IBS), Seoul National University, Seoul 08826, Republic of Korea

[2] School of Chemical and Biological Engineering, and Institute of Chemical Process, Seoul National University, Seoul 08826, Republic of Korea

[3]Institute of Engineering Research, College of Engineering, Seoul National University, Seoul 08826, Republic of Korea

[4]Advanced Institute of Convergence Technology, Seoul National University, Suwon-si, Gyeonggi-do 16229, Republic of Korea

[5]Current Address: Samsung Advanced Institute of Technology, Samsung Electronics, Suwon 16678, Republic of Korea

[6]Current Address: Bio-MAX/N-Bio, Institute of BioEngineering, Seoul National University, Seoul, Republic of Korea

[7]Current Address: Department of Materials Science and Engineering, Northwestern University, Evanston, Illinois 60208, United States

* These authors jointly supervised this work: T. Hyeon (thyeon@snu.ac.kr) & J. Park (jungwonpark@snu.ac.kr)

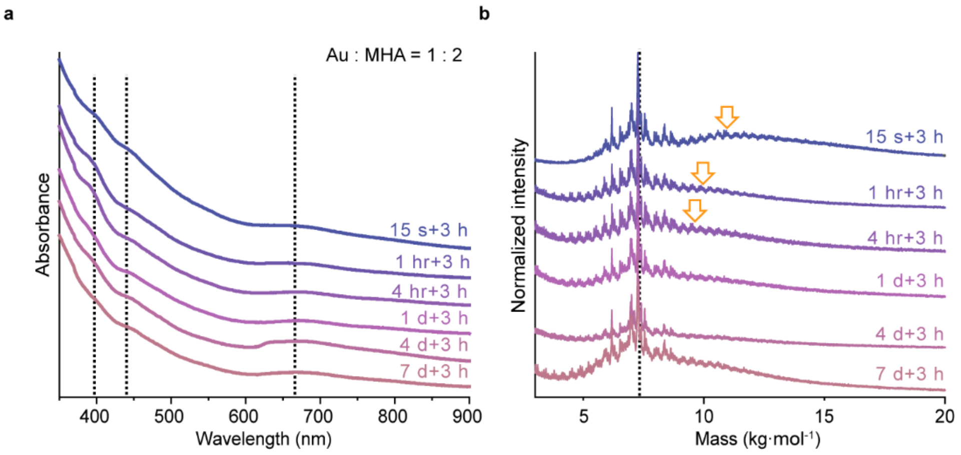

Supplementary Fig. 1. Au NCs synthesized under different conditions of first reduction time. a, UV-Vis spectra, and **b**, MALDI-TOF mass spectra (in the linear mode) of Au NCs synthesized under each conditions of different first reduction time (from short first reduction time, blue to long first reduction time, purple and pink). The ratio of Au precursor to MHA is fixed as 1:2, and second reduction time under all conditions is fixed as 3 h. Dashed lines indicate the existence of Au_{25} NCs in both **a** and **b**. Arrows in **b** indicate the mass of Au NCs bigger than $Au_{25}(MHA)_{18}$. The data of 15 s+3 h (blue color) and 7 d+3 h (pink color) are identical with those in **Fig. 1.** Source data are provided as a Source Data file.

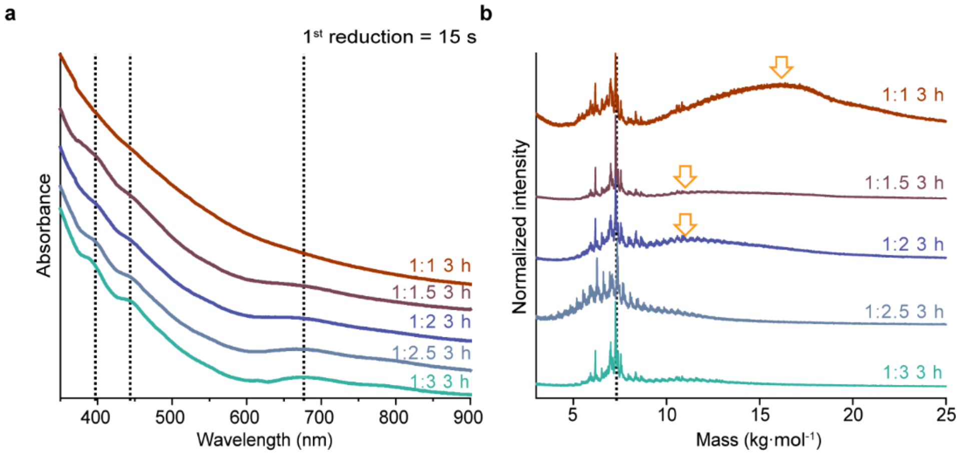

Supplementary Fig. 2. Au NCs synthesized under different conditions of Au to MHA ratio. **a**, UV-Vis spectra, and **b**, MALDI-TOF mass spectra (in the linear mode) of Au NCs synthesized under each conditions of different ratio of Au precursor to MHA (from Au : MHA = 1:1, brown to Au : MHA = 1:3, blue and turquoise). The first reduction time is fixed as 15 s, and second reduction time under all conditions is fixed as 3 h. Dashed lines indicate the existence of Au_{25} NCs in both **a** and **b**. Arrows in **b** indicate the mass of Au NCs bigger than $Au_{25}(MHA)_{18}$. The data of 1:1 3 h (brown color), 1:2 3 h (blue color), and 1:3 3 h (turquoise color) are identical with those in **Fig. 1**. Source data are provided as a Source Data file.

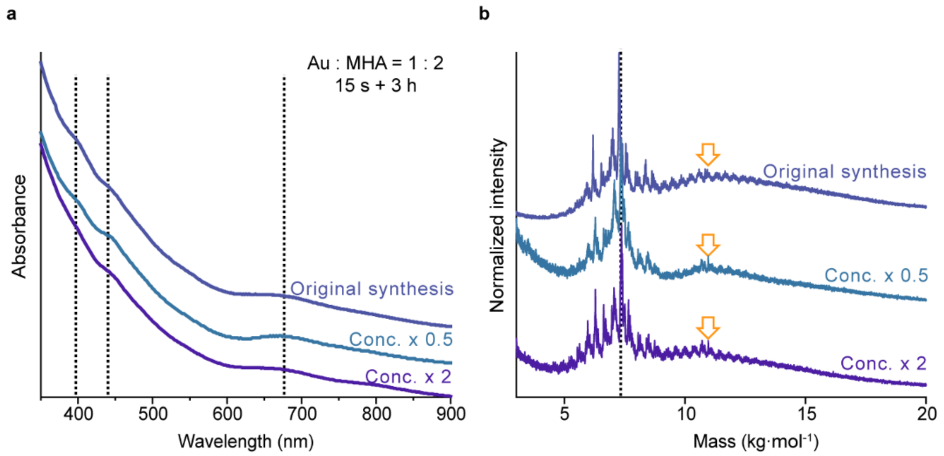

Supplementary Fig. 3. Au NCs synthesized under different conditions of initial reactant concentration. **a**, UV-Vis spectra, and **b**, MALDI-TOF mass spectra (in the linear mode) of Au NCs synthesized under three conditions of different initial reactant concentration (half of original concentration, cobalt, twice of original concentration, indigo). The ratio of Au precursor to MHA is fixed as 1:2, the first reduction time is fixed as 15 s, and second reduction time under all conditions is fixed as 3 h. Dashed lines indicate the existence of Au_{25} NCs in both **a** and **b**. Arrows in **b** indicate the mass of Au NCs bigger than $Au_{25}(MHA)_{18}$. The data of original synthesis is identical with those in **Fig 1.** Source data are provided as a Source Data file.

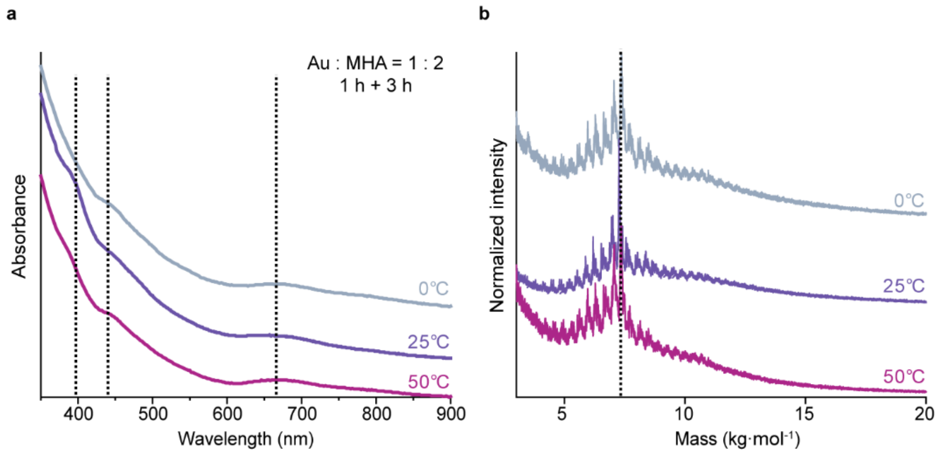

Supplementary Fig. 4. Au NCs synthesized under different conditions of temperature. a, UV-Vis spectra, and **b**, MALDI-TOF mass spectra (in the linear mode) of Au NCs synthesized under three conditions of different temperature (0 °C, baby blue, 50 °C, magenta). The ratio of Au precursor to MHA is fixed as 1:2, the first reduction time is fixed as 1 h, and second reduction time under all conditions is fixed as 3 h. Dashed lines indicate the existence of Au_{25} NCs in both **a** and **b**. The data of 25°C is identical with those in **Supplementary Fig. 1**. Source data are provided as a Source Data file.

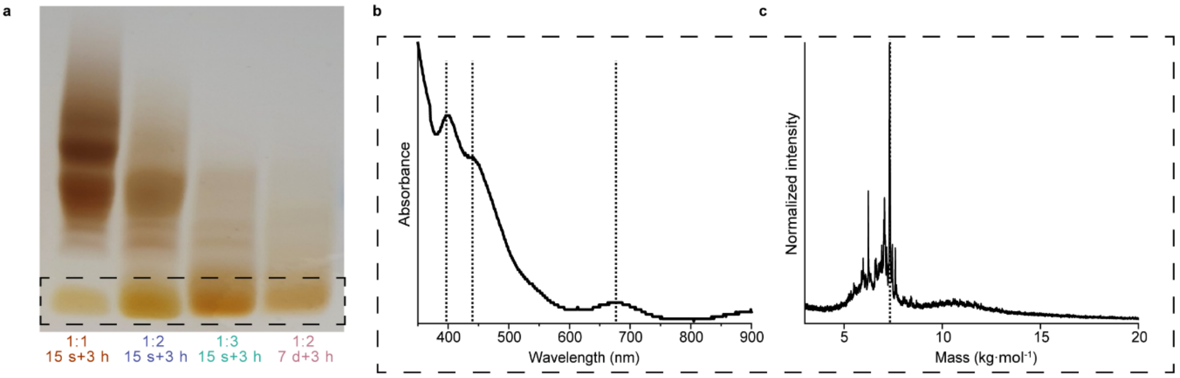

Supplementary Fig. 5. PAGE experiments of Au NCs in Fig. 1. a, Digital photograph of PAGE gel separating Au NCs obtained under different conditions in **Fig. 1**. Dashed rectangle indicates the location of $Au_{25}(MHA)_{18}$. **b**, UV-Vis spectra, and **c**, MALDI-TOF mass spectra (in the linear mode) of $Au_{25}(MHA)_{18}$ NCs acquired from the part of dashed rectangle from (A). Dashed lines indicate the existence of Au_{25} in both **b** and **c**. Source data are provided as a Source Data file.

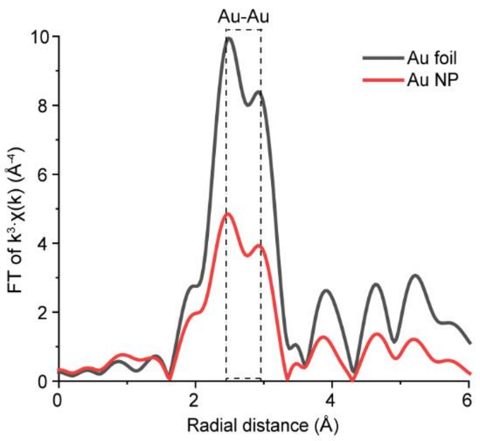

Supplementary Fig. 6. References of Au-Au peaks in EXAFS. Experimental EXAFS spectra of Au foil (black) and Au NP (red) to verify the position of Au-Au peaks. The K-range window was 3-11 $Å^{-1}$. Source data are provided as a Source Data file.

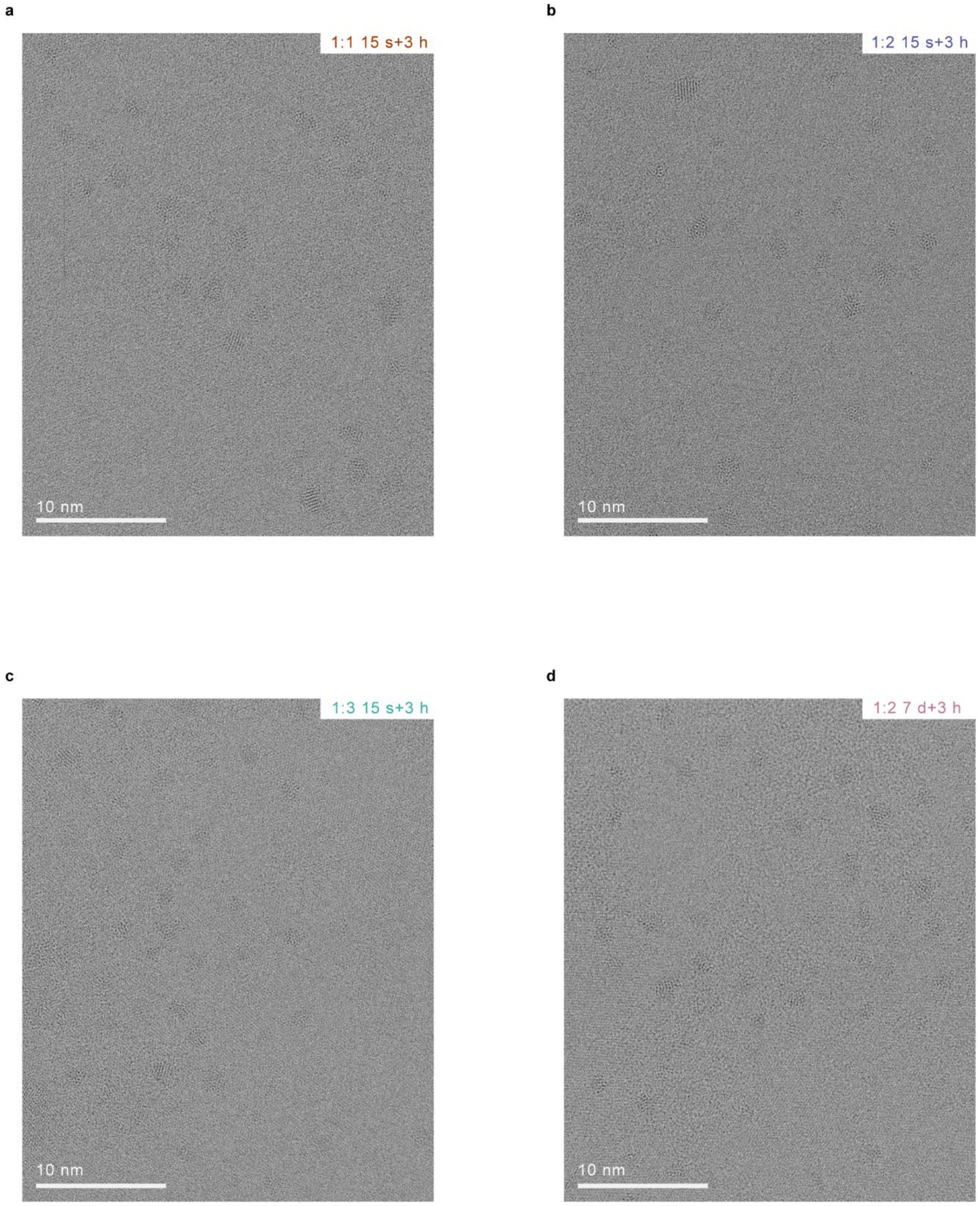

Supplementary Fig. 7. Original Cs-TEM images of Fig. 1d-g. Original Cs-TEM images of Au NCs under **a**, 1:1 15 s+3 h condition, **b**, 1:2 15 s+3 h condition, **c**, 1:3 15 s+3 h condition, and **d**, 1:2 7 d+3 h condition, which are matched with the data in **Fig. 1d-g** respectively.

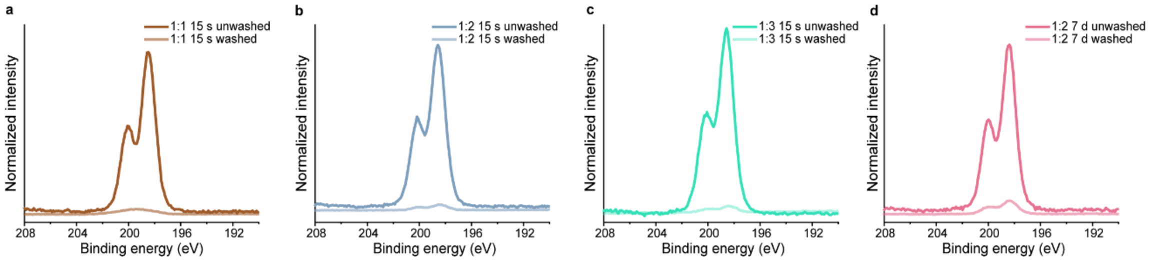

Supplementary Fig. 8. Cl 2*p* XPS spectra of Au-MHA complexes in Fig. 2. Cl 2*p* XPS spectra of unwashed (bold color) and washed (pale color) samples of Au-MHA complexes under each conditions. The colors of all data are identical with those in **Fig. 2**. Source data are provided as a Source Data file.

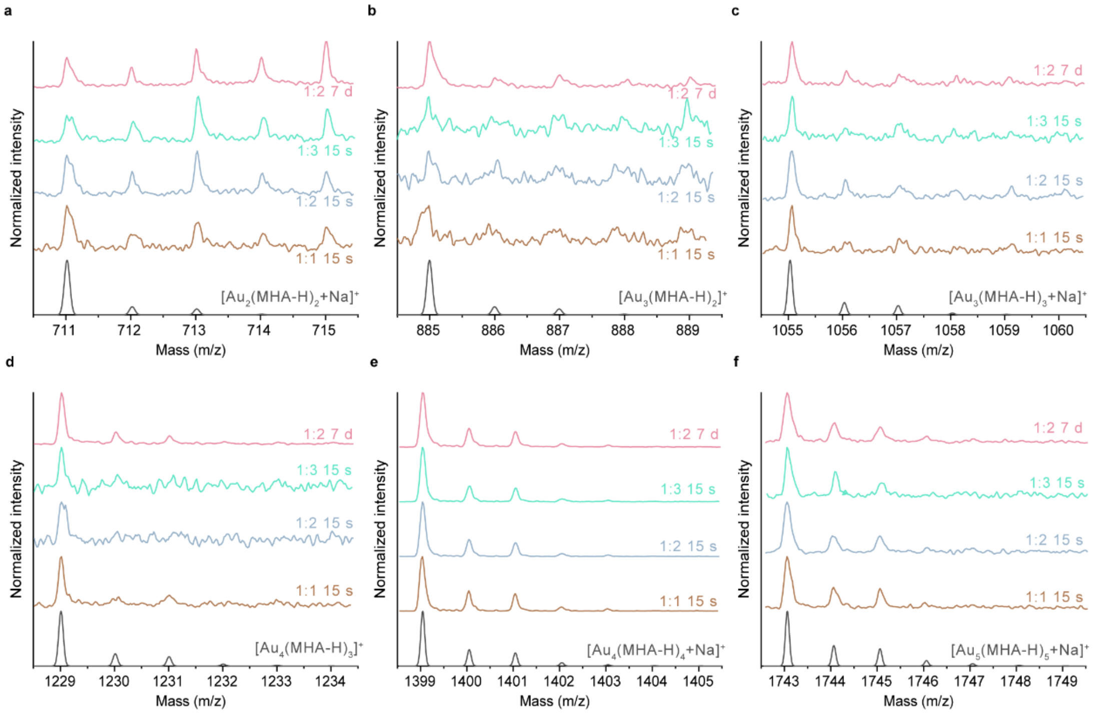

Supplementary Fig. 9. Isotope calculation of several Au-MHA complexes under different conditions. Experimental and calculated (black) mass spectra of all complexes detected in the samples of Au precursors in **Fig. 2**. All colors are corresponding to those in **Fig. 2a**. Experimental spectra of $[Au_2(MHA\text{-}H)_2\text{+}Na]^+$ complexes under the four conditions are convoluted with mass spectra of DHB matrix. Source data are provided as a Source Data file.

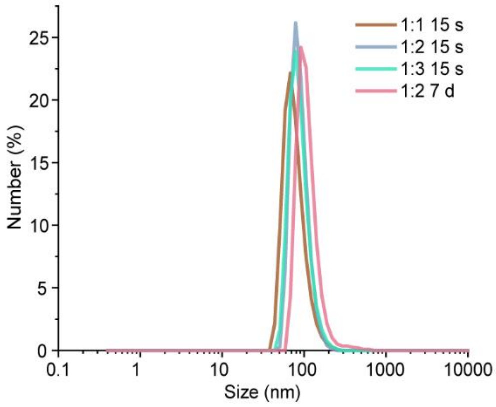

Supplementary Fig. 10. DLS data of Au-MHA complexes under different conditions. DLS spectrum of aggregated Au-MHA complexes under each conditions. The colors of samples are identical with the data in **Fig. 2**. Source data are provided as a Source Data file.

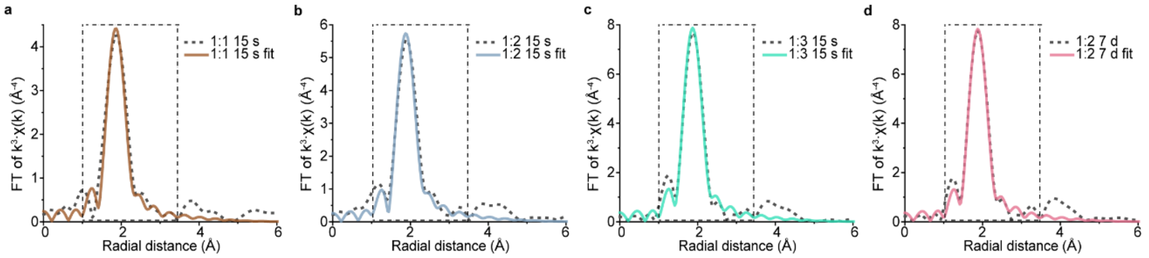

Supplementary Fig. 11. Fitting of EXAFS data of Au-MHA complexes in real space. Experimental (dot) and fitted (solid) EXAFS spectra of Au-MHA complexes from **Fig. 2d-g** in real space under **a**, 1:1 15 s condition, **b**, 1:2 15 s condition, **c**, 1:3 15 s condition, and **d**, 1:2 7 d condition. Dashed rectangles indicate the R-space range of 1-3.5 Å where the fitting was done. The colors of experimental data are identical with those in **Fig. 2**. Source data are provided as a Source Data file.

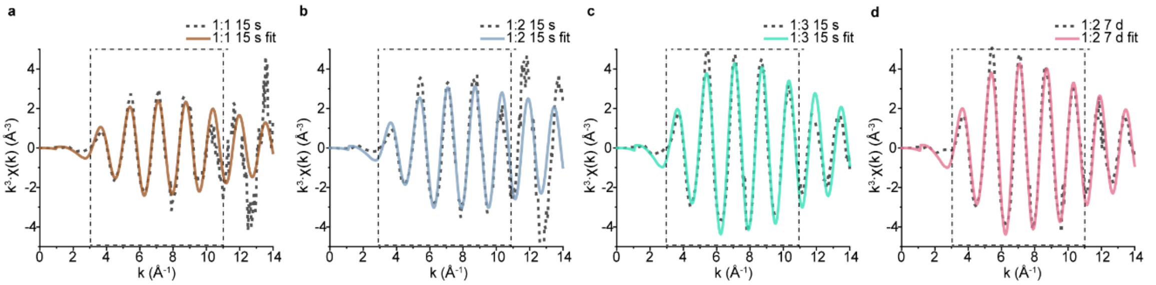

Supplementary Fig. 12. Fitting of EXAFS data of Au-MHA complexes in k-space. Experimental (dot) and fitted (solid) EXAFS spectra of Au-MHA complexes from **Fig. 2d-g** in k-space under **a**, 1:1 15 s condition, **b**, 1:2 15 s condition, **c**, 1:3 15 s condition, and **d**, 1:2 7 d condition. Dashed rectangles indicate the k-space range of 3-11 $Å^{-1}$ where the fitting was done. The colors of experimental data are identical with those in **Fig. 2**. Source data are provided as a Source Data file.

Supplementary Table 1. Au EXAFS fitting results for the Au-MHA complexes in each synthetic conditions. The amplitude reduction factor, S_0^2, was derived from the EXAFS fitting of standard Au foil to 1.33. The K-range window was 3-11 Å^{-1} and the fitting was done in an R-space that ranged in 1-3.5 Å. The parameters about Au-S bonds are calculated from the fitted spectrum of EXAFS with Au_2S as a reference.

Sample	Path	R (Å)	Coordination number	Debye-Waller factor, σ^2 (Å^2)
Au : MHA = 1:1 15 s	Au-S	1.87	0.93	0.0029
Au : MHA = 1:2 15 s			1.10	0.0020
Au : MHA = 1:3 15 s			1.76	0.0033
Au : MHA = 1:2 7 d			1.80	0.0036

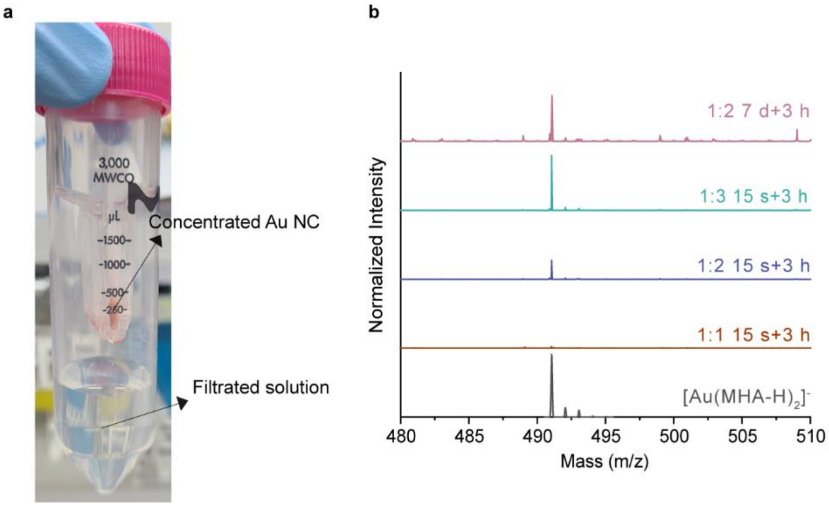

Supplementary Fig. 13. Separation of $[Au(MHA)_2]^-$ complex from the Au NC solution in Fig. 1. a, Digital photograph of separated solution of side product (bottom) and concentrated Au NC solutions in **Fig. 1**, Experimental and calculated (black) ESI mass spectra of $[Au(MHA)_2]^-$ complex in the filtrated solution under the different conditions. All colors are corresponding to those in **Fig. 1**. Source data are provided as a Source Data file.

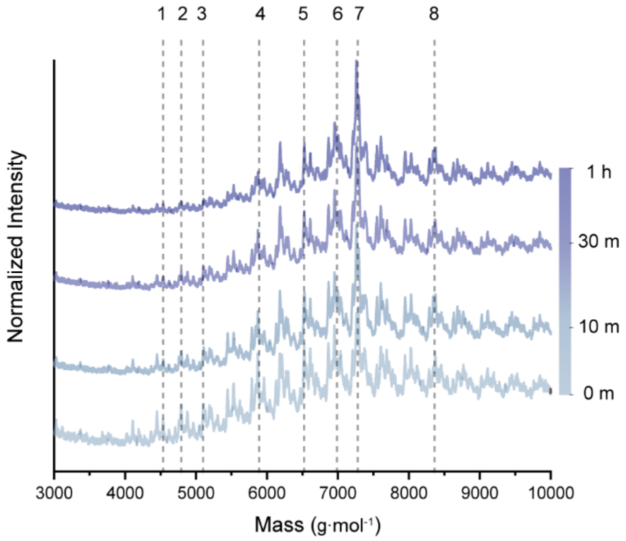

	Au NC
1	$Au_{15}(MHA)_{13}$ ($2e^-$)
2	$[Au_{16}(MHA)_{13}]^-$ ($2e^-$)
3	$[Au_{17}(MHA)_{14}]^-$ ($4e^-$)
4	$[Au_{20}(MHA)_{15}]^-$ ($6e^-$)
5	$[Au_{23}(MHA)_{16}]^-$ ($8e^-$)
6	$Au_{23}(MHA)_{17}$ ($6e^-$)
7	$[Au_{25}(MHA)_{18}]^-$ ($8e^-$)
8	$[Au_{29}(MHA)_{20}]^-$ ($10e^-$)

Supplementary Fig. 14. Identification of intermediate Au NCs during the second reduction under the condition in Fig. 3d~f. (Left) Zoom-in mass spectra of intermediate Au NCs in **Fig. 3d~f**. (from 0 m of second reduction, light blue to 1 h of second reduction, bold blue) Dashed lines indicate the intermediate Au NCs labeled with numbers. (Right) Table of the molecular formulas labeled NCs in mass spectra with the number of free valence electrons in parentheses. Source data are provided as a Source Data file.

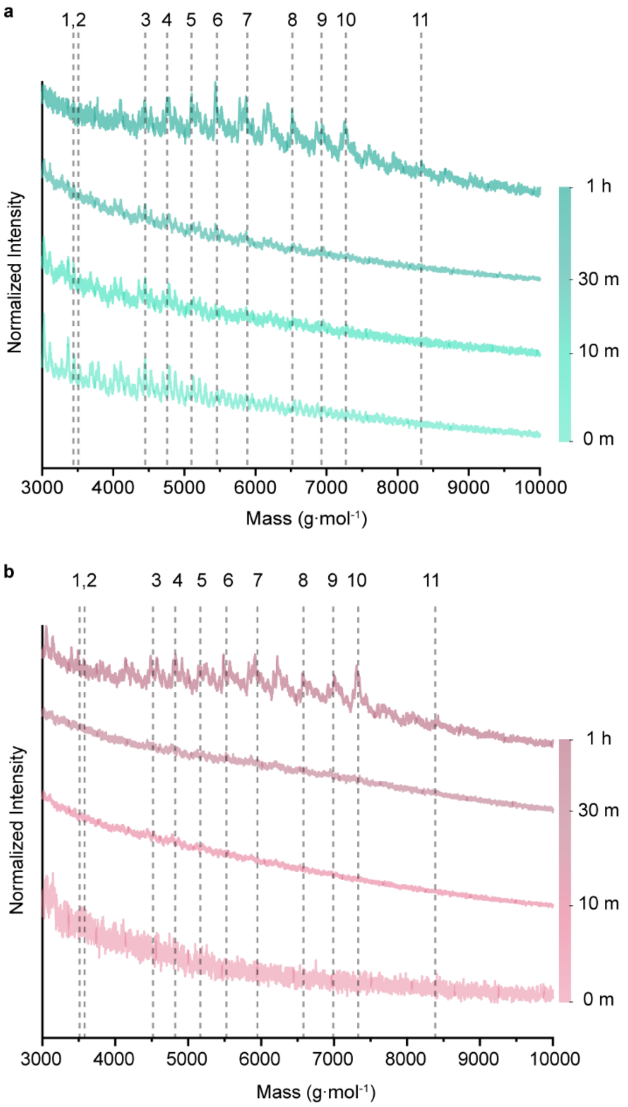

c

	Au NC
1	$Au_{10}(MHA)_{10}$ (0e$^-$)
2	$Au_{11}(MHA)_{9}$ (2e$^-$)
3	$Au_{15}(MHA)_{13}$ (2e$^-$)
4	$[Au_{16}(MHA)_{13}]^-$ (4e$^-$)
5	$[Au_{17}(MHA)_{14}]^-$ (4e$^-$)
6	$Au_{18}(MHA)_{14}$ (4e$^-$)
7	$[Au_{20}(MHA)_{15}]^-$ (6e$^-$)
8	$[Au_{23}(MHA)_{16}]^-$ (8e$^-$)
9	$Au_{24}(MHA)_{18}$ (6e$^-$)
10	$[Au_{25}(MHA)_{18}]^-$ (8e$^-$)
11	$[Au_{29}(MHA)_{20}]^-$ (10e$^-$)

Supplementary Fig. 15. Identification of intermediate Au NCs during the second reduction under the conditions in Fig. 4. a,b, Zoom-in mass spectra of intermediate Au NCs in **Fig. 4a~c** and **Fig. 4d~f**, respectively. Dashed lines indicate the intermediate Au NCs labeled with numbers. **c**, Table of the molecular formulas labeled NCs in **a**,**b** with the number of free valence electrons in parentheses. Source data are provided as a Source Data file.

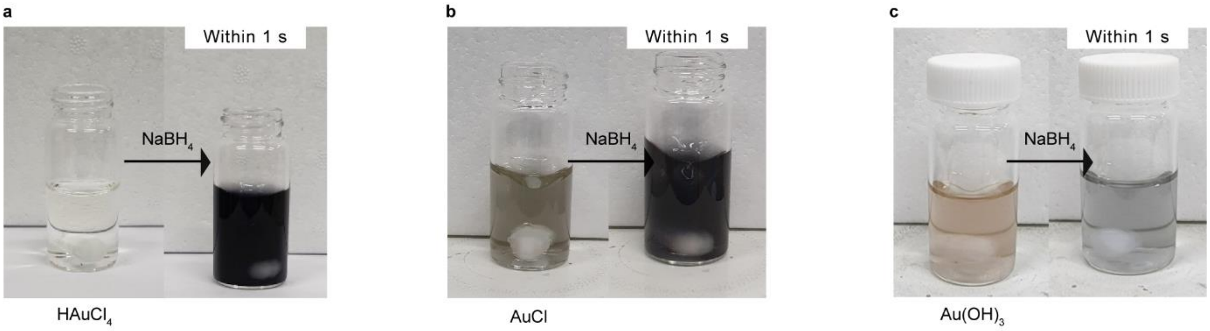

Supplementary Fig. 16. Reduction of Au salts coordinated with non-sulfur anions. a, Digital photographs of $HAu^{III}Cl_4$ before (left) and after reacting with $NaBH_4$ (right). **b**, Digital photographs of $Au^{I}Cl$ before (left) and after reacting with $NaBH_4$ (right). **c**, Digital photographs of $Au^{III}(OH)_3$ before (left) and after reacting with $NaBH_4$ (right). Source data are provided as a Source Data file.

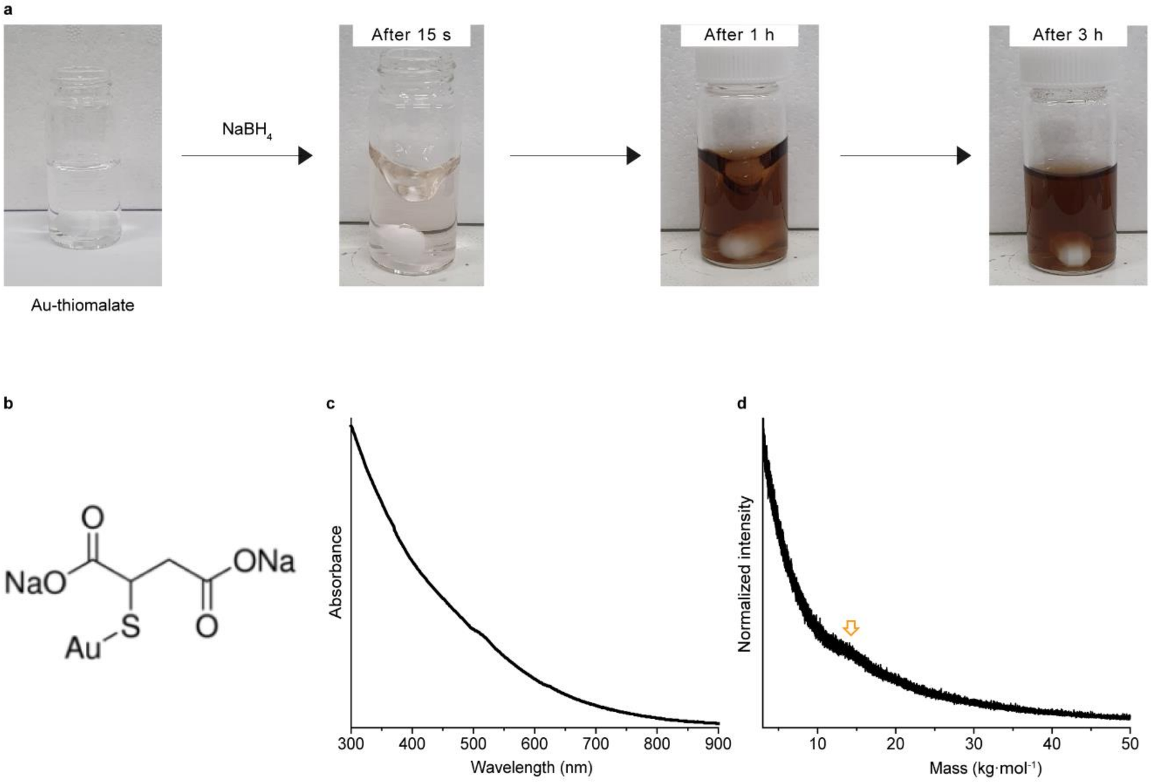

Supplementary Fig. 17. Reduction of Au^{I}-thiomalate. a, Digital photographs of Au^{I}-thiomalate before (left first) and after the injection of $NaBH_4$ with reaction time of 15 s (second), 1 hour (third) and 3 h (fourth). **b**, Chemical structure of Au^{I}-thiomalate used in the experiment. **c**, UV-Vis absorption spectra and **d**, MALDI-TOF mass spectra (in the linear mode) of Au NCs synthesized from the second reduction of Au^{I}-thiomalate after 3 h. An arrow in **d** indicates the mass of produced Au NCs from the reduction of Au^{I}-thiomalate. Source data are provided as a Source Data file.

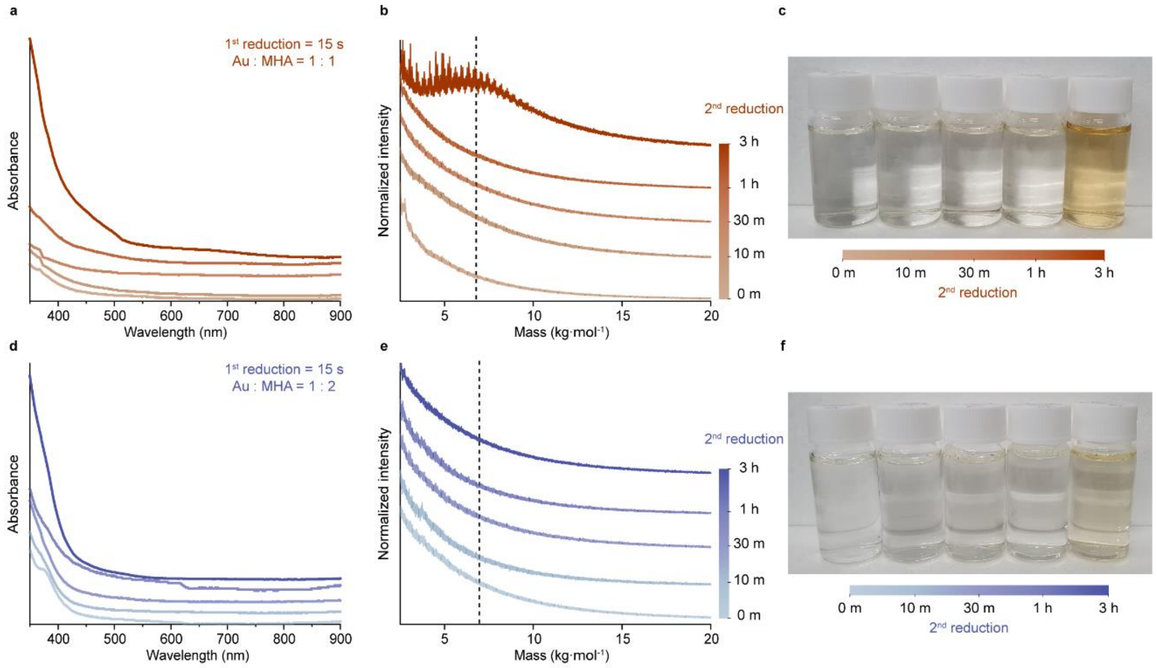

Supplementary Fig. 18. Reduction of washed complexes under the same condition with Fig. 3. a, UV-Vis absorption spectra, **b**, MALDI-TOF mass spectra in the linear mode, and **c**, Digital photo of synthesized Au NCs during the second reduction of washed Au-MHA precursors under 1:1 15 s condition. **d**, UV-Vis absorption spectra, **e**, MALDI-TOF mass spectra in the linear mode, and **f**, Digital photo of synthesized Au NCs during the second reduction of washed Au-MHA precursors under 1:2 15 s condition. Dashed line in mass spectra indicates the mass of Au_{25} NCs. Source data are provided as a Source Data file.

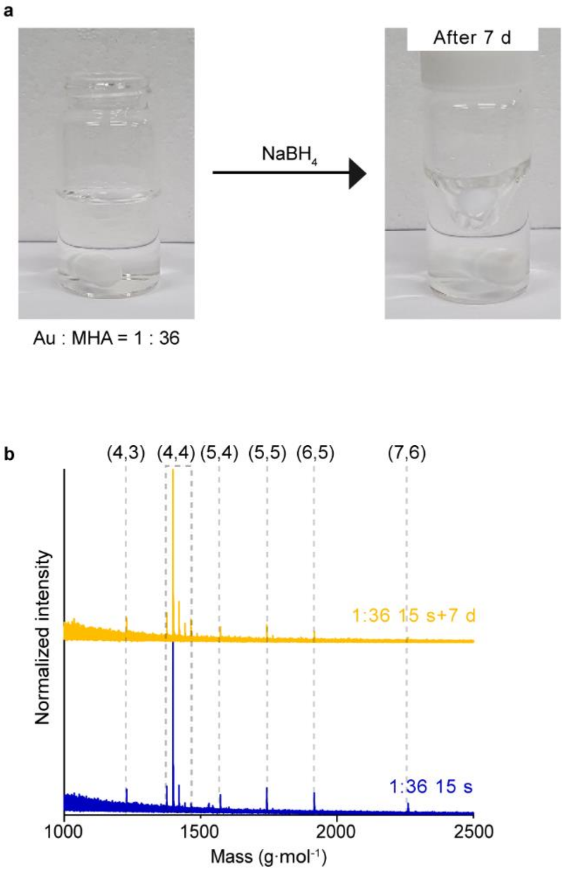

Supplementary Fig. 19. Reduction of focused $Au_4(MHA)_4$ complexes. a, Digital photographs of Au-MHA complexes synthesized under the conditions of Au to MHA ratio as 1:36 before (left) and after injection of $NaBH_4$ with reaction time of 7 days (right). **b**, MALDI-TOF mass spectra (in the reflector mode) of samples in **a**. (before $NaBH_4$ injection, blue, after $NaBH_4$ injection, yellow) Dashed lines indicate the Au-MHA complexes matched with the number of Au and MHAs in parentheses, and dashed rectangles indicate the $Au_4(MHA)_4$ family with Na^+ adducts. Source data are provided as a Source Data file.

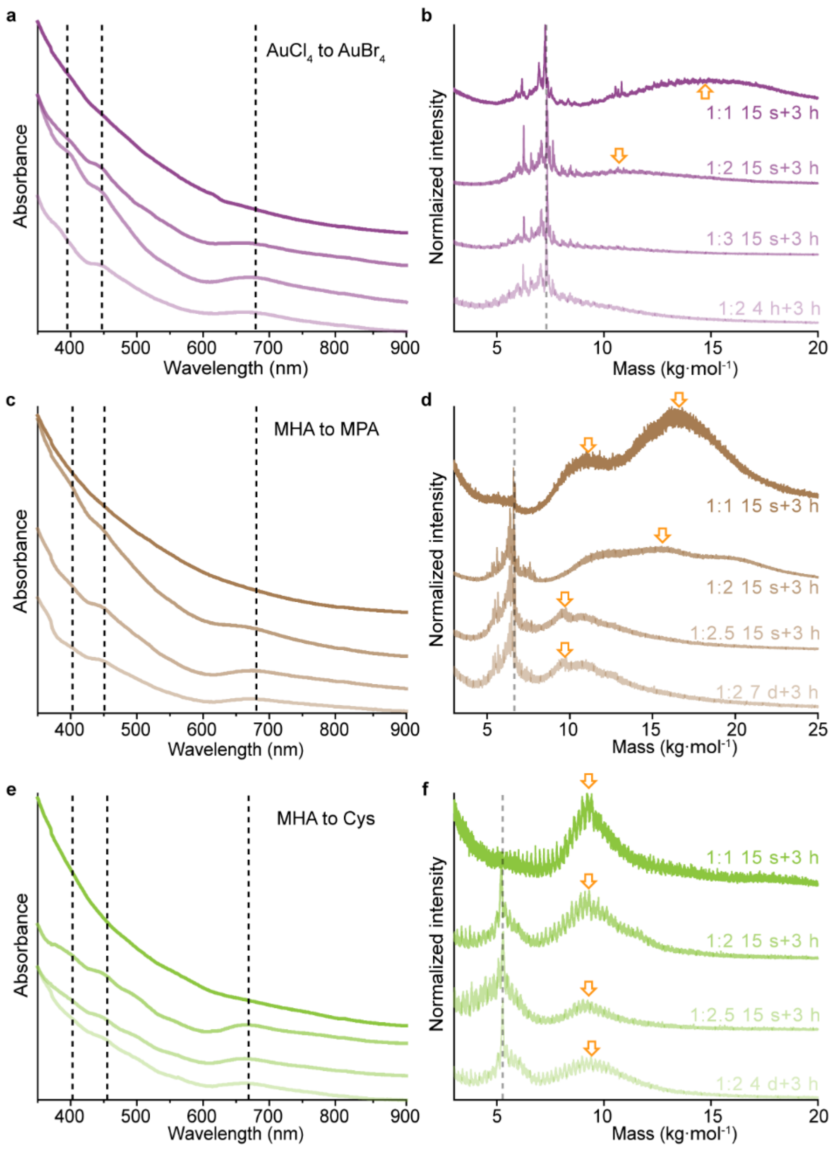

Supplementary Fig. 20. Synthesis of homogeneous Au NCs by controlling complex formation with different precursors or ligands. a, UV-Vis absorption spectra, and **b**, MALDI-TOF mass spectra (in the linear mode) of Au NCs synthesized from $HAuBr_4$ with different conditions of the first reduction (less extent of complex formation, bold violet, more extent of complex formation, pale violet). **c**, UV-Vis absorption spectra, and **d**, MALDI-TOF mass spectra (in the linear mode) of MPA-protected Au NCs with different conditions of the first reduction (less extent of complex formation, bold rust, more extent of complex formation, pale rust). **e**, UV-Vis absorption spectra, and **f**, MALDI-TOF mass spectra (in the linear mode) of Cys-protected Au NCs with different conditions of the first reduction (less extent of complex formation, bold pea green, more extent of complex formation, pale pea green). Dashed lines in UV-Vis absorption spectra and mass spectra indicate the evidence of Au_{25} NCs. Arrows in mass spectra indicate the Au NCs larger than $Au_{25}(SR)_{18}$. Source data are provided as a Source Data file.

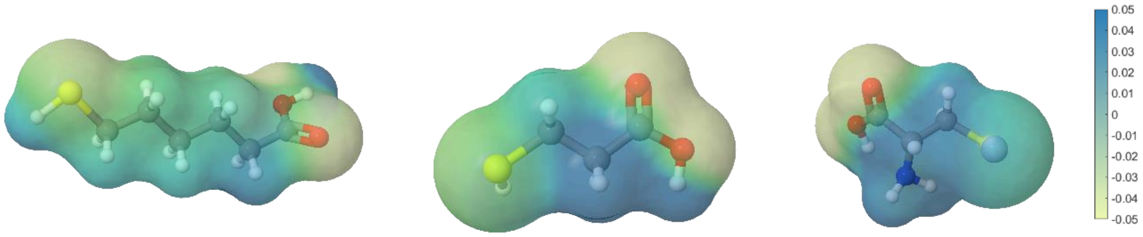

Supplementary Fig. 21. Chemical structures of different thiol ligands. Chemical structures of **a**, MHA, **b**, MPA, and **c**, Cys ligands with electrostatic potential (ESP) maps (black, C; white, H; red, O; blue, N; yellow, S). The color of ESP maps becomes ivory as electron density of atom is rich, and becomes blue as electron density of atom is deficient. ESP potential is calculated in Hatree atomic unit.

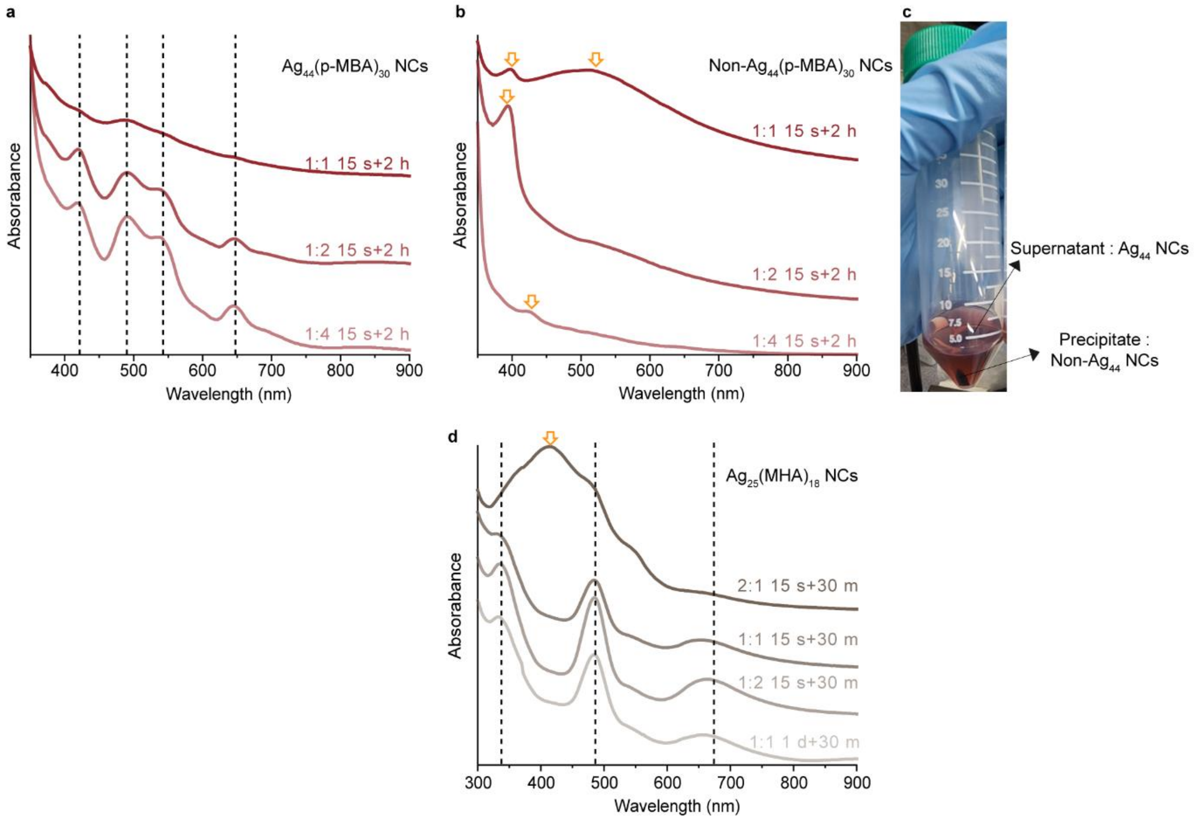

Supplementary Fig. 22. Synthesis of two types of Ag NCs with different extent of complex formation. **a**, UV-Vis absorption spectra of Ag_{44}(*p*-MBA)$_{30}$ NCs and **b**, separated non-Ag_{44} NCs under the different conditions (less extent of complex formation, bold burgundy, more extent of complex formation, light burgundy). Dashed lines in **a**, indicate the evidence of Ag_{44}(*p*-MBA)$_{30}$ NCs. Arrows in **b** indicate the distinct peaks of non-Ag_{44} NCs. The data of 1:4 15 s+2 h in **a** is identical with that in **Fig. 5a.** **c**, Digital photograph of separated Ag_{44} NCs in supernatant and precipitated non-Ag_{44} by one simple step of centrifugation. **d**, UV-Vis absorption spectra of $Ag_{25}(MHA)_{18}$ NCs under the different conditions (less extent of complex formation, bold gray, more extent of complex formation, light gray). Dashed lines in **d** indicate the evidence of $Ag_{25}(MHA)_{18}$ NCs. An arrow in **d** indicates the LSPR peaks of metallic Ag NPs. The data of 1:2 15 s+30 min **d** is identical with that in **Fig. 5b.** Source data are provided as a Source Data file.

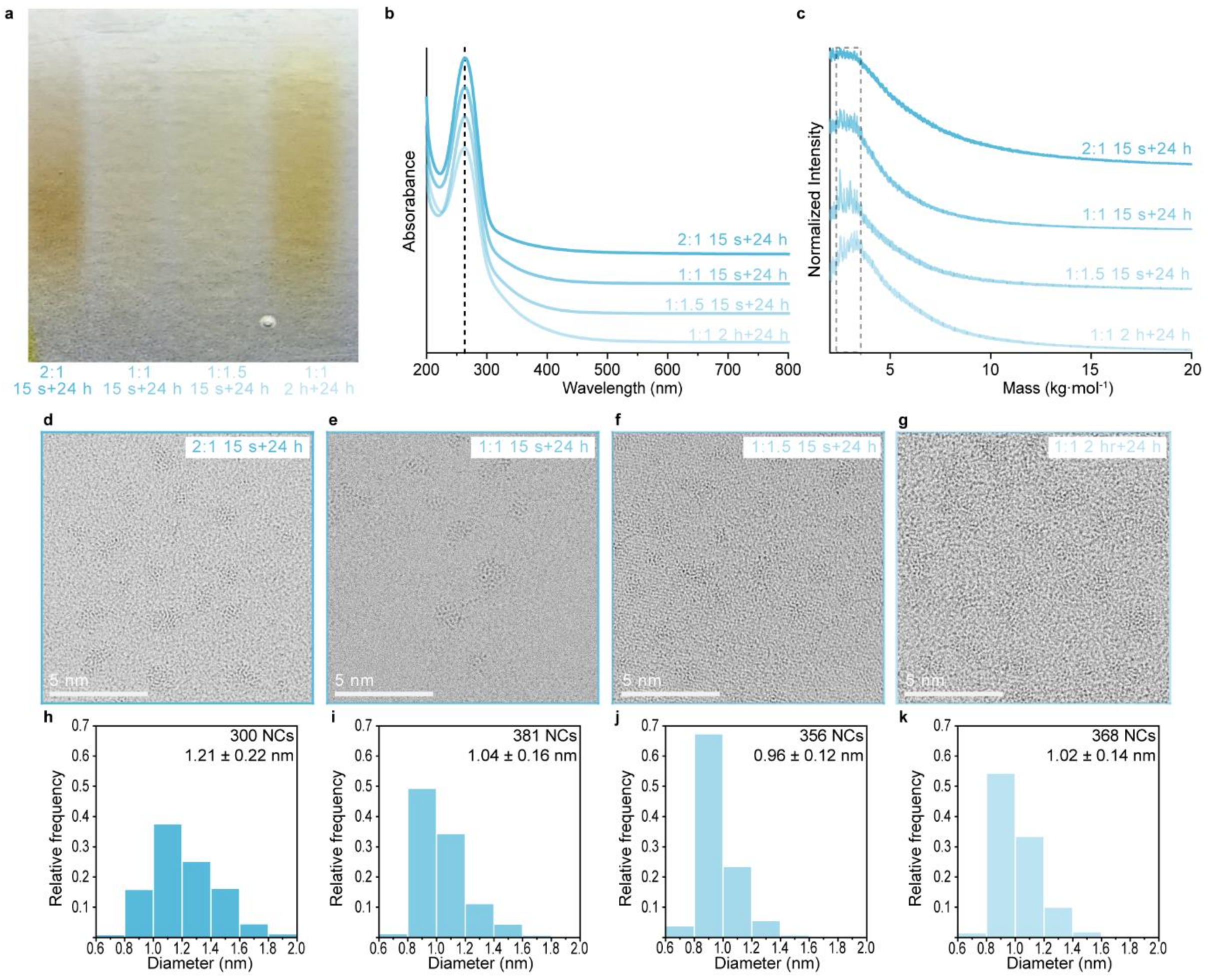

Supplementary Fig. 23. Synthesis of Pt NCs with different extent of complex formation. **a**, Digital photograph of PAGE gel separating Pt NCs obtained the under different conditions. **b**, UV-Vis spectra, and **c**, MALDI-TOF mass spectra (in the linear mode) of Pt NCs obtained under the different conditions (less extent of complex formation, bold sky blue, more extent of complex formation, light sky blue). A dashed line in **b** indicates the distinct absorption of quasi-molecular Pt NCs. A dashed rectangle in **c** indicates the mass of ultrasmall-sized Pt NCs. **d-g**, Representative C_s-TEM images of Pt NCs under different conditions. **h-k**, Size distribution of the Pt NCs from **d-g**, respectively. Total number of the counted NCs, average and standard deviation in measured diameter of the NCs are also listed in histograms. The data of 1:1.5 15 s+24 h are identical with those in **Fig. 5c,h**. Source data are provided as a Source Data file.

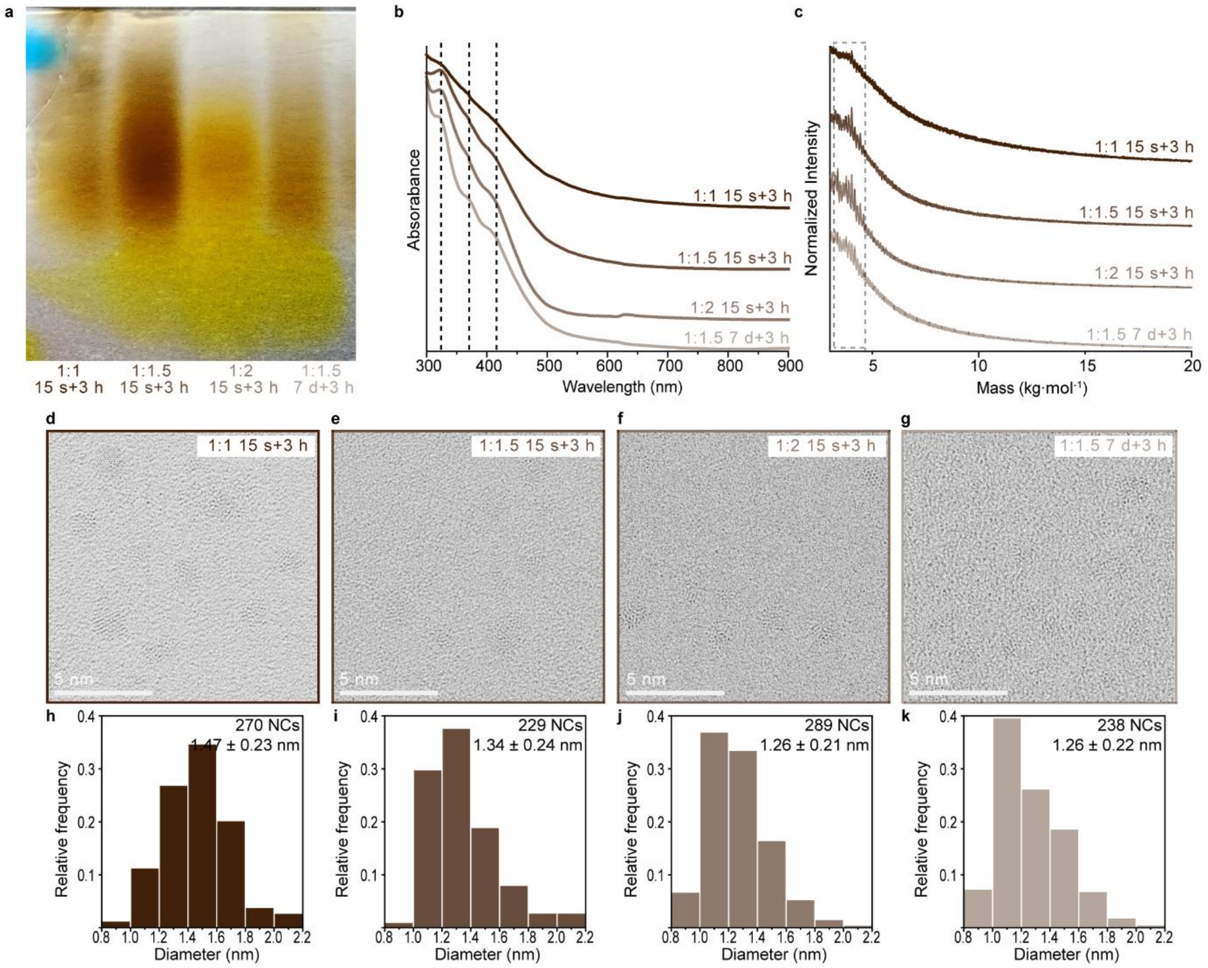

Supplementary Fig. 24. Synthesis of Pd NCs with different extent of complex formation. **a**, Digital photograph of PAGE gel separating Pd NCs obtained the under different conditions. **b**, UV-Vis spectra, and **c**, MALDI-TOF mass spectra (in the linear mode) of Pd NCs obtained under the different conditions (less extent of complex formation, bold auburn, more extent of complex formation, light auburn). Dashed lines in **b** indicate the distinct absorption of quasi-molecular Pd NCs. A dashed rectangle in **c** indicates the mass of ultrasmall-sized Pd NCs. **d-g**, Representative C_s-TEM images of Pd NCs under different conditions. **h-k**, Size distribution of the Pd NCs from **d-g**, respectively. Total number of the counted NCs, average and standard deviation in measured diameter of the NCs are also listed in histograms. The data of 1:2 15 s+3 h are identical with those in **Fig. 5d,i**. Source data are provided as a Source Data file.

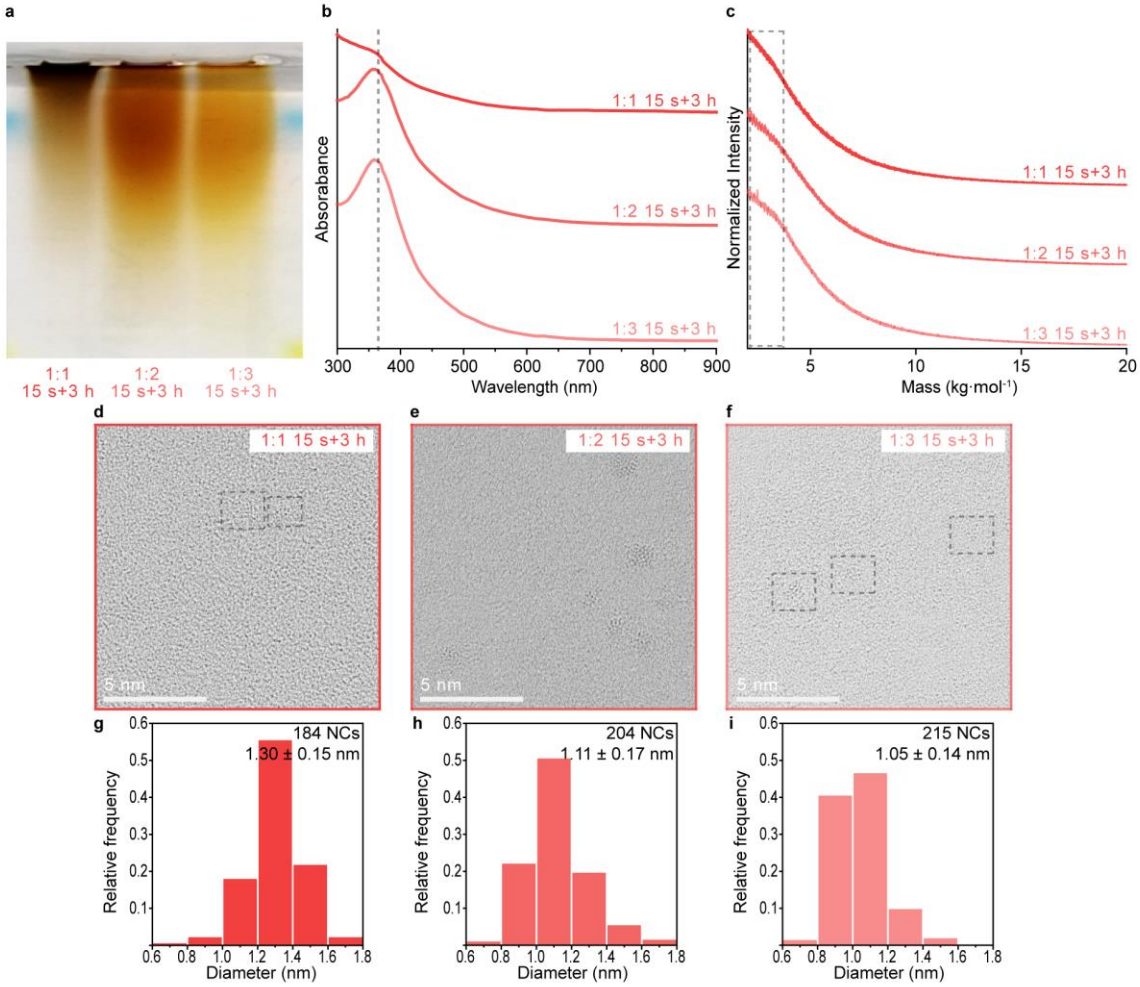

Supplementary Fig. 25. Synthesis of Rh NCs with different extent of complex formation. **a**, Digital photograph of PAGE gel separating Rh NCs obtained the under different conditions. **b**, UV-Vis spectra, and **c**, MALDI-TOF mass spectra (in the linear mode) of Rh NCs obtained under the different conditions (less extent of complex formation, bold red, more extent of complex formation, light red). A dashed line in **b** indicates the distinct absorption of quasi-molecular Rh NCs. A dashed rectangle in **c** indicates the mass of ultrasmall-sized Rh NCs. **d-f**, Representative C_s-TEM images of Rh NCs under different conditions. **g-i**, Size distribution of the Rh NCs from **d-f**, respectively. Dashed rectangle in **d**,**f** indicate the existence of ultrasmall-sized Rh NCs. Total number of the counted NCs, average and standard deviation in measured diameter of the NCs are also listed in histograms. The data of 1:3 15 s+3 h are identical with those in **Fig. 5e,j.** Source data are provided as a Source Data file.

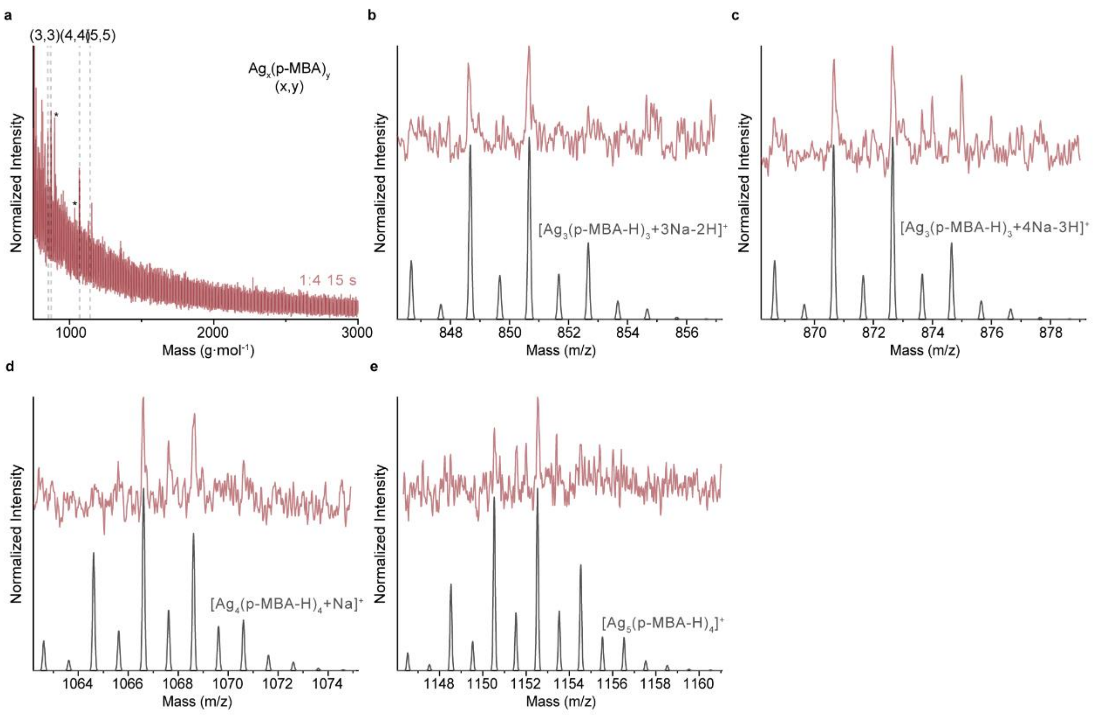

Supplementary Fig. 26. Isotope calculation of Ag-(*p*-MBA) complexes under the condition of Fig. 5a,f. a, MALDI-TOF mass spectra (in the reflector mode) of Ag-(*p*-MBA) complexes under the condition of 1:4 15 s. Dashed lines indicate the Ag-(*p*-MBA) complexes matched with the number of Ag and *p*-MBA in parentheses. Mass peaks marked with asterisks originate from the DHB matrix. **b-e**, Experimental and calculated (black) mass spectra of the specific complexes in **a**. The color of all experimental data is identical with that of **Fig. 5a,f**. Source data are provided as a Source Data file.

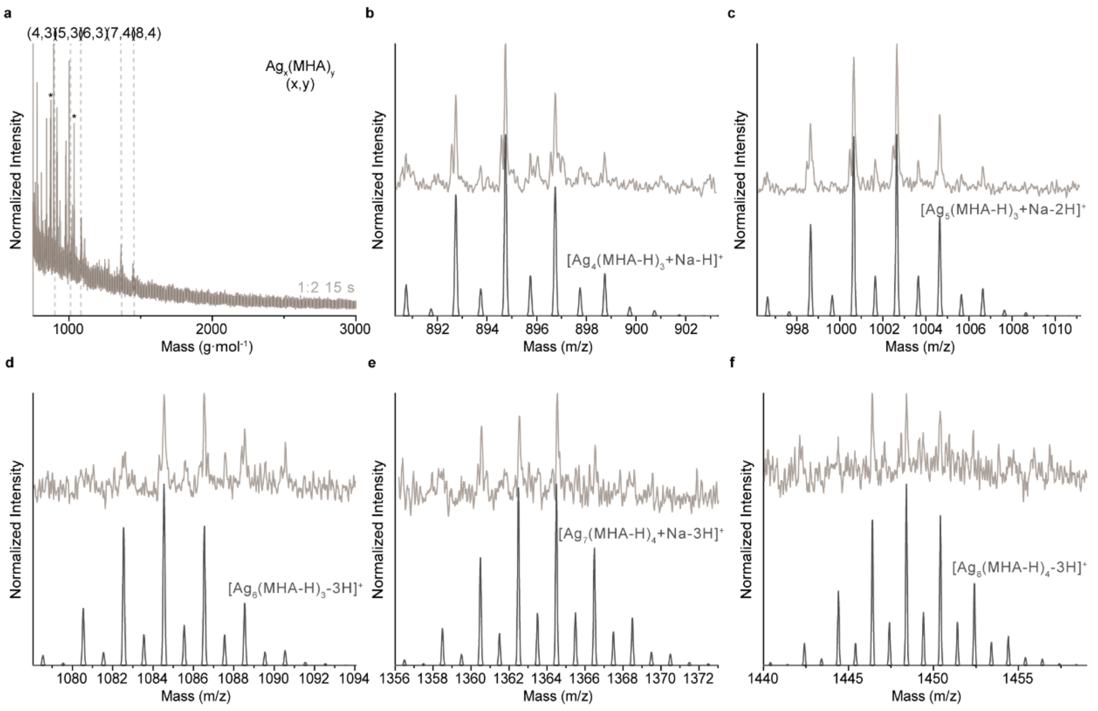

Supplementary Fig. 27. Isotope calculation of Ag-MHA complexes under the condition of Fig. 5b,g. a, MALDI-TOF mass spectra (in the reflector mode) of Ag-MHA complexes under the condition of 1:2 15 s. Dashed lines indicate the Ag-MHA complexes matched with the number of Ag and MHA in parentheses. Mass peaks marked with asterisks originate from the DHB matrix. **b-f**, Experimental and calculated (black) mass spectra of the specific complexes in **a**. The color of all experimental data is identical with that of **Fig. 5b,g**. Source data are provided as a Source Data file.

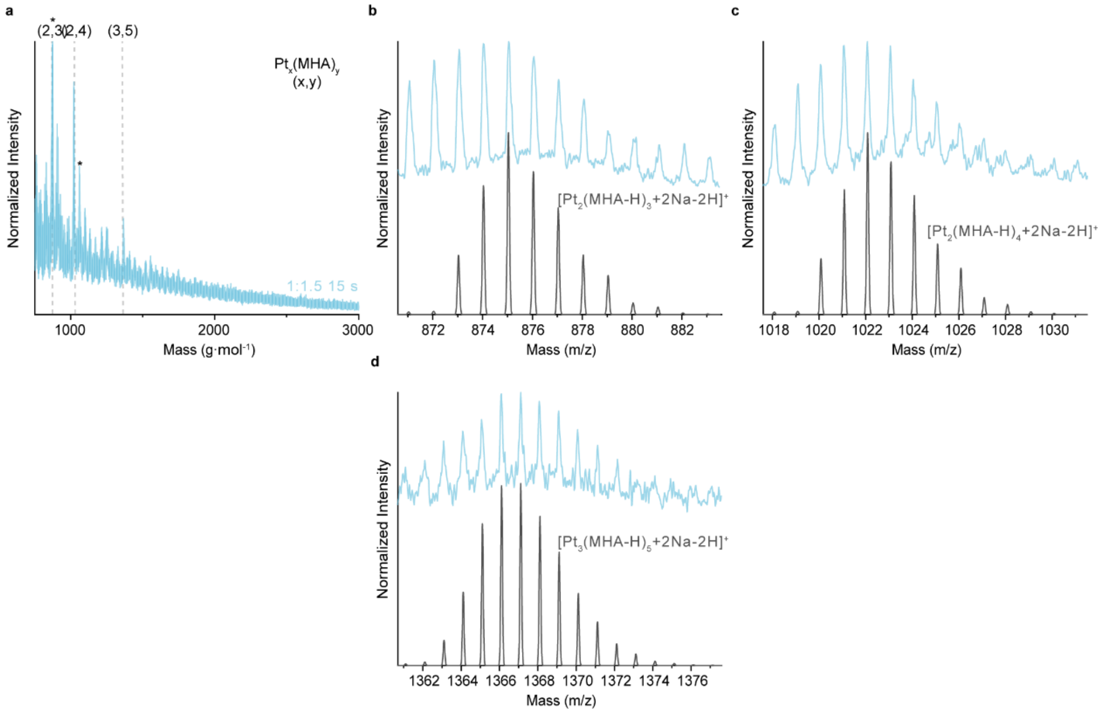

Supplementary Fig. 28. Isotope calculation of Pt-MHA complexes under the condition of Fig. 5c,h. a, MALDI-TOF mass spectra (in the reflector mode) of Pt-MHA complexes under the condition of 1:1.5 15 s. Dashed lines indicate the Pt-MHA complexes matched with the number of Pt and MHA in parentheses. Mass peaks marked with asterisks originate from the DHB matrix. **b-d**, Experimental and calculated (black) mass spectra of the specific complexes in **a**. Experimental spectra of $[Pt_2(MHA\text{-}H)_3\text{+}2Na\text{-}2H]^+$ complexes in **b** is convoluted with mass spectra of DHB matrix. The color of all experimental data is identical with that of **Fig. 5c,h**. Source data are provided as a Source Data file.

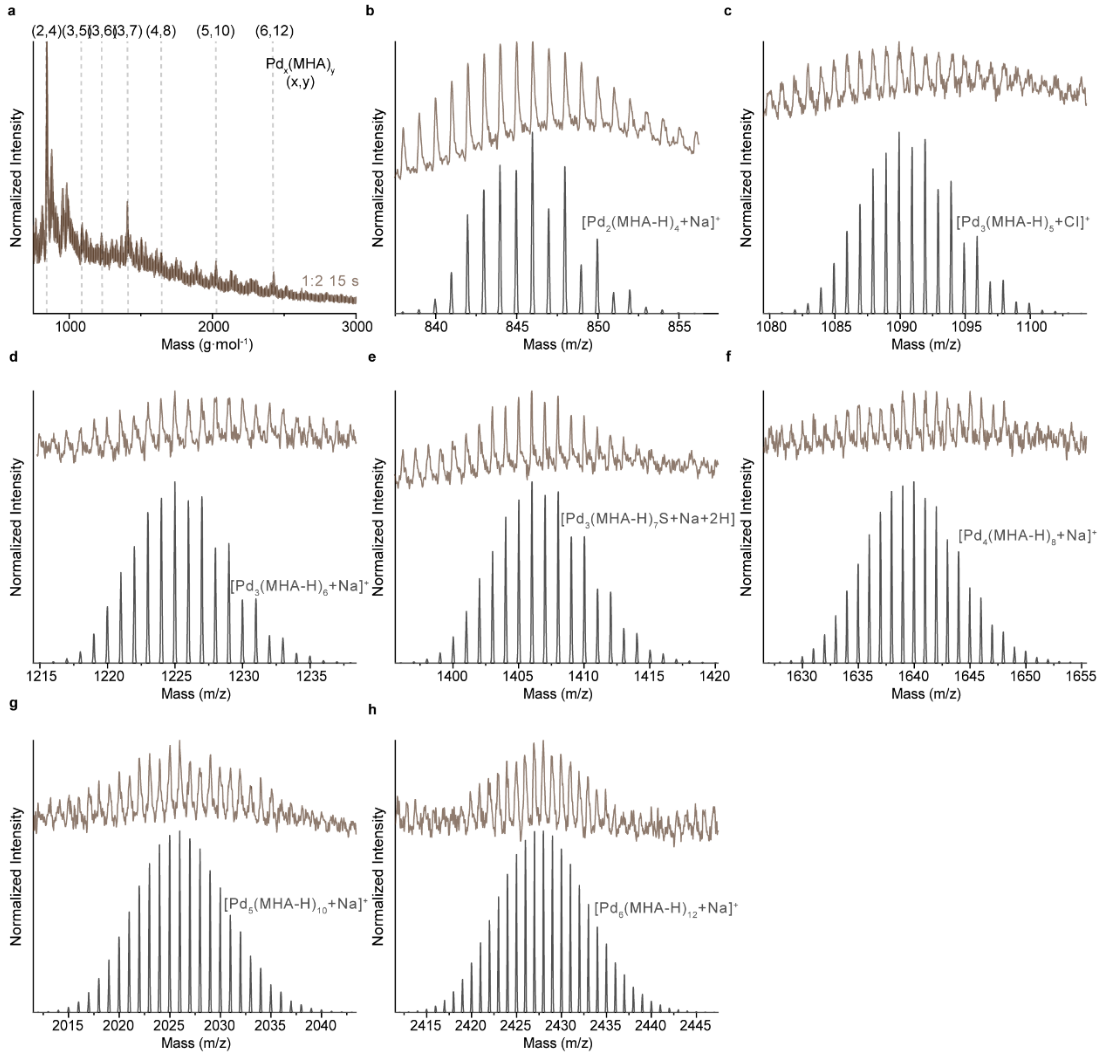

Supplementary Fig. 29. Isotope calculation of Pd-MHA complexes under the condition of Fig. 5d,i. a, MALDI-TOF mass spectra (in the reflector mode) of Pd-MHA complexes under the condition of 1:2 15 s. Dashed lines indicate the Pd-MHA complexes matched with the number of Pd and MHA in parentheses. **b-h**, Experimental and calculated (black) mass spectra of the specific complexes in **a**. The color of all experimental data is identical with that of **Fig. 5d,i**. Source data are provided as a Source Data file.

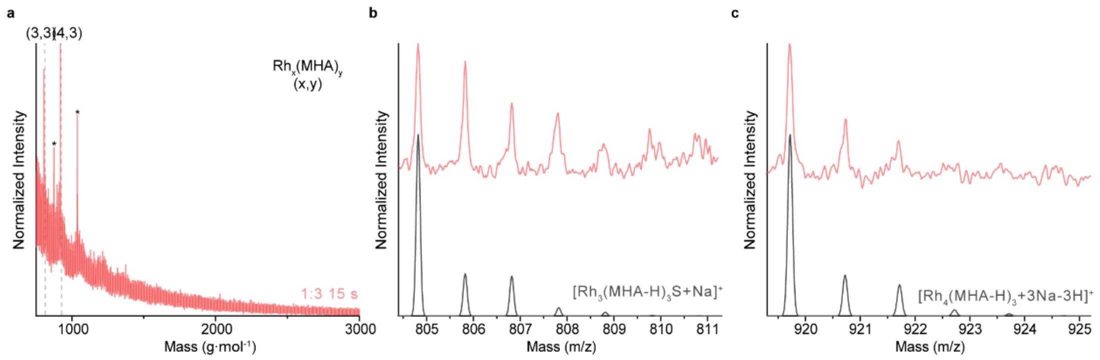

Supplementary Fig. 30. Isotope calculation of Rh-MHA complexes under the condition of Fig. 5e,j. a, MALDI-TOF mass spectra (in the reflector mode) of Rh-MHA complexes under the condition of 1:3 15 s. Dashed lines indicate the Rh-MHA complexes matched with the number of Rh and MHA in parentheses. Mass peaks marked with asterisks originate from the DHB matrix. **b**,**c**, Experimental and calculated (black) mass spectra of the specific complexes in **a**. The color of all experimental data is identical with that of **Fig. 5e,j**. Source data are provided as a Source Data file.

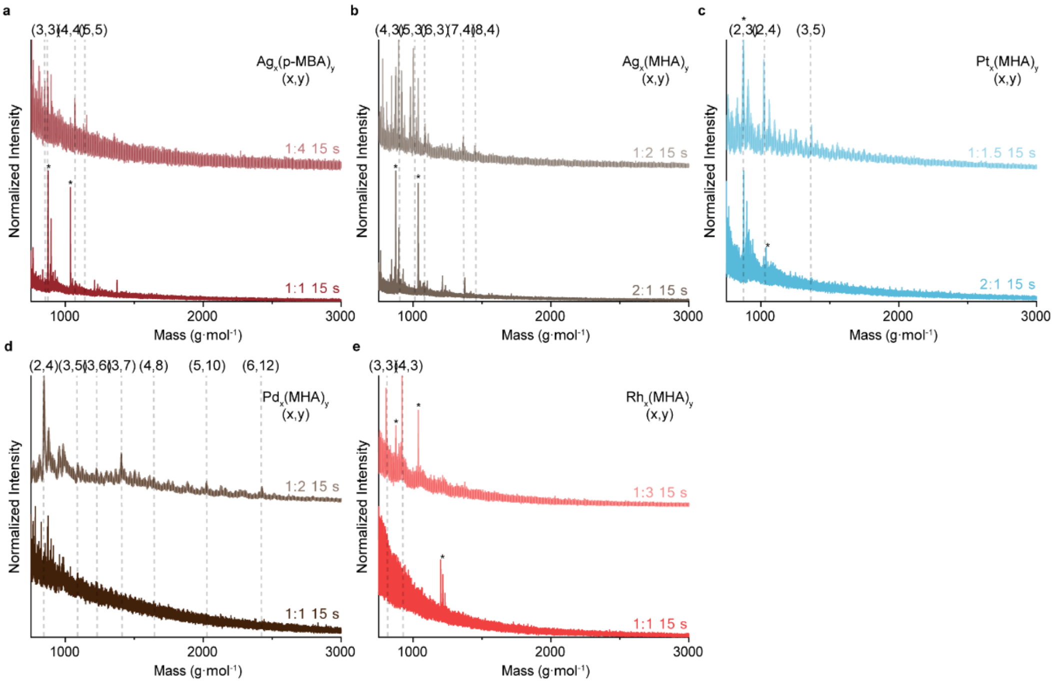

Supplementary Fig. 31. Differences in the extent of metal-thiolate complex formation under the different conditions. MALDI-TOF mass spectra (in the reflector mode) of **a**, Ag-(*p*-MBA) complexes, **b**, Ag-MHA complexes, **c**, Pt-MHA complexes, **d**, Pd-MHA complexes, and **e**, Rh-MHA complexes under the different conditions. Dashed lines indicate the metal-thiolate complexes matched with the number of the metal and MHA in parentheses. Mass peaks marked with asterisks originate from the DHB matrix. The bold colors are corresponding to those in **Supplementary Fig. 22-25**, and the data with pale color are identical with those in **Supplementary Fig. 26-30**. Source data are provided as a Source Data file.

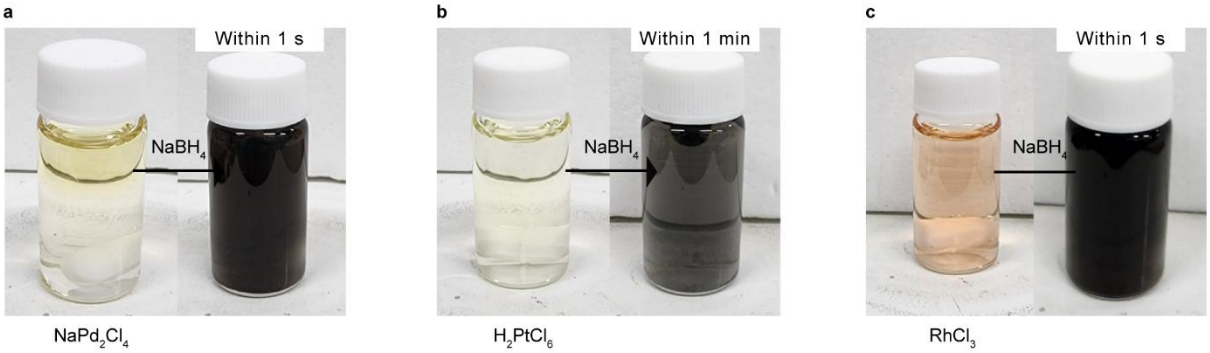

Supplementary Fig. 32. Reduction of metal salts coordinated with non-sulfur anions. a, Digital photographs of Na_2PdCl_4 before (left) and after reacting with $NaBH_4$ (right). **b**, Digital photographs of H_2PtCl_6 before (left) and after reacting with $NaBH_4$ (right). **c**, Digital photographs of $RhCl_3$ before (left) and after reacting with $NaBH_4$ (right).
